# Implementation of Early Intervention Services (OPUS) in Denmark and results of 20 year follow-up of the OPUS trial

**DOI:** 10.1192/j.eurpsy.2023.80

**Published:** 2023-07-19

**Authors:** M. Nordentoft

**Affiliations:** Mental Health Center Copenhagen, Copenhagen University Hospital, Hellerup, Denmark

## Abstract

**Abstract: Background:**

The OPUS trial is the largest randomized controlled trial (RCT) testing early intervention services with 20-years of follow-up among individuals with a first episode of psychosis in the schizophrenia spectrum.

**Methods:**

A total of 547 individuals with first episode psychosis in the schizophrenia spectrum were included into the OPUS I trial between January 1998 - December 2000 and allocated to either two years of early intervention services or treatment as usual. Clinical and trained staff, blinded to the original treatment allocation, performed the five, ten and 20-year follow-up assessments.

The early intervention service consisted of two years of assertive community treatment including social skill training, psychoeducation and family involvement delivered by a multi-disciplinary team (staff patient ratio 1:10). The standard treatment was based on the available community mental health treatment (1:20 –1:30).

**Results:**

A total of 164 participants (30%) of 547 were interviewed at the 20-year follow-up. No significant differences were found between the early intervention group (OPUS-group) compared to the Treatment As Usual group (TAU-group) on global functional levels, psychotic and negative symptom scores after 20 years. Likewise, no differences was found ten to 20-years after randomization between the OPUS-group and TAU-group on days of psychiatric hospitalizations (Incidence Rate Ratio (IRR), 1.202, 95% CI 0.733 - 1.997, P=0.46), or number of outpatient contacts (IRR: 1.197, 95% CI 0.889 - 1.612, P=0.24). Of the entire cohort, 40% were in symptom remission and 18% were in clinical recovery at the 20-year follow-up. The mortality rate 20 years after randomization was 13.1% in the OPUS-group and 15.1% in the TAU group, P=.47.

**Conclusions and Relevance:**

New initiatives are needed to maintain the positive outcomes achieved after two years of early intervention services

**Disclosure of Interest:**

None Declared

